# The Usefulness of Determining Neutrophil Gelatinase-Associated Lipocalin Concentration Excreted in the Urine in the Evaluation of Cyclosporine A Nephrotoxicity in Children with Nephrotic Syndrome

**DOI:** 10.1155/2016/6872149

**Published:** 2016-12-26

**Authors:** Ewa Gacka, Marcin Życzkowski, Rafał Bogacki, Andrzej Paradysz, Lidia Hyla-Klekot

**Affiliations:** ^1^Department of Pediatric Nephrology, Center for Pediatrics and Oncology, Chorzów, Poland; ^2^Department of Urology, School of Medicine with Division of Dentistry in Zabrze, Medical University of Silesia, Katowice, Poland

## Abstract

*Introduction.* The use of cyclosporine (CsA) in the treatment of nephrotic syndrome (NS) contributed to a significant reduction in the amount of corticosteroids used in therapy and its cumulative side effects. One of the major drawbacks of CsA therapy is its nephrotoxicity. Prolonged CsA treatment protocols require sensitive, easily available, and simple to measure biomarkers of nephrotoxicity. NGAL is an antibacterial peptide, excreted by cells of renal tubules in response to their toxic or inflammatory damage.* Aim of the Study.* The aim of this study was to assess the suitability of the NGAL concentration in the urine as a potential biomarker of the CsA nephrotoxicity.* Material and Methods.* The study was performed on a group of 31 children with NS treated with CsA. The control group consisted of 23 children diagnosed with monosyptomatic enuresis. The relationship between NGAL excreted in urine and the time of CsA treatment, concentration of CsA in blood serum, and other biochemical parameters was assessed.* Results.* The study showed a statistically significant positive correlation between urine NGAL concentration and serum triglycerides concentration and no correlation between C0 CsA concentration and other observed parameters of NS. The duration of treatment had a statistically significant influence on the NGAL to creatinine ratio.* Conclusions.* NGAL cannot be used alone as a simple CsA nephrotoxicity marker during NS therapy. Statistically significant correlation between NGAL urine concentration and the time of CsA therapy indicates potential benefits of using this biomarker in the monitoring of nephrotoxicity in case of prolonged CsA therapy.

## 1. Introduction

Since the 1980s calcineurin inhibitors have been the basis of immunosuppressive therapy in the prevention of transplant rejection and in the treatment of various autoimmune diseases. A significant limitation to their long-term use is a specifically defined nephrotoxicity. The nephrotoxic action of calcineurin inhibitors depends on genetically determined drug pharmacodynamics and demonstrates a dependence on their serum concentrations and on the duration of the therapy [1, 2]. In the practice of a paediatric nephrologist, cyclosporine A is used for the management of steroid-resistant nephrotic syndrome, minimal change nephrotic syndrome, FSGS, and secondary nephropathies [3]. The introduction of CsA into the management of chronic glomerulonephritis in 1988 contributed to a significant limitation of chronic steroid therapies in children and their late, cumulative adverse effects. The remission rate of steroid-dependent nephrotic syndrome following cyclosporine A usage is estimated at 80–100%, and, in the case of steroid-resistant nephrotic syndrome, the value amounts to 30% [4]. Cyclosporine-dependence, that is, a recurrence of proteinuria, amounts to 25% when the dosage is reduced and rises up to 80% during attempts to discontinue the treatment, which is a considerable problem associated with CsA therapies [5]. Cyclosporine-dependence forces long-term usage of the medicine, which, despite strict monitoring of the concentration, increases the risk of nephrotoxicity. The detection of an early stage of toxicity could be the basis for important therapeutic decisions, for example, CsA conversion to mycophenolate mofetil.

Ischaemic nephron injury, caused by afferent arteriole constriction, develops in early stages of cyclosporine-induced nephropathy [6]. The drug's adverse effect initially involves the proximal tubule cells which are subject to oedema and vacuolization. Inflammatory infiltration in renal interstitium is also an early sign of CsA toxicity [7].

The most precise way to assess the nephrotoxic effect of CsA is a kidney biopsy. A histopathological analysis reveals features of arteriolopathy (hyalinization of nephron afferent arterioles), band-like interstitial fibrosis, and ischaemic atrophy of the renal tubules. The detection of changes typical for the CsA toxicity in a renal biopsy specimen attests to a considerable advancement of the process and has a nature of chronic and progressing remodelling of the nephron and tubulointerstitial compartment [7].

Numerous studies have reported that ischaemic, osmotic, or toxic injury (e.g., following cisplatin use) of the epithelial cells of the proximal tubules induces the synthesis of a large amount of neutrophil gelatinase-associated lipocalin (NGAL) by the cells of the distal tubules and intercalated cells of the arcuate tubules [8, 9].

NGAL, which is called “renal troponin,” is a sensitive marker of acute renal injury [10]. It is proven that a considerable increase in its serum concentration and its increased urinary excretion occur in acute and chronic renal failure, severe urinary tract infections, after cardiac surgery connected with acute kidney injury (AKI), in contrast-induced nephropathy and secondary to toxic effects of xenobiotics [11, 12].

NGAL excreted in urine is a sum of the protein filtered in the renal glomeruli (the majority of this pool of protein is absorbed by the proximal tubule), synthesized de novo by cells of the nephron loop thick limb and arcuate renal tubules, as well as a pool of protein produced by neutrophils and macrophages that infiltrate the organ during an ongoing inflammation [13–15].

Thus, an increase in NGAL concentration in urine may indicate the impairment of the reabsorption of this protein by the proximal tubule cells, increased NGAL synthesis by the distal parts of nephron as a reaction to inflammatory or ischaemic injury, and activation of immune cells in the renal parenchyma and migrant cells [13, 16].

In the available literature there are single reports concerning the usefulness of determining the level of NGAL in urine for the detection of the early stages of cyclosporine A nephrotoxicity in children chronically treated with this drug due to glomerulopathy with nephrotic syndrome [17].

## 2. Aim of the Study

The aim of the study was to evaluate the usefulness of determining the NGAL urine concentration as a potential biomarker of cyclosporine A nephrotoxicity.

## 3. Material and Methods

The examined group consisted of 31 patients, children and adolescents, at the age of 3–17 (mean 9.2), including 19 boys and 12 girls. The patients were treated in the Department and Outpatient Clinic of Paediatric Nephrology of Chorzow Centre for Paediatrics and Oncology due to steroid-dependent or steroid-resistant nephrotic syndrome. Characteristics of the examined group are showed in Table 1. The children with normal kidney function (GFR > 90 ml/min/l.73 m^2^) were included in the study, after exclusion of active foci of infection, UTIs, urinary tract anomalies, and liver diseases.

All patients received cyclosporine A for at least 3 months. The mean duration of the therapy was 26.5 months. The daily dose ranged from 3 to 6 mg/kg of the body weight.

During the entire treatment, the serum drug concentration was systematically monitored, with C0 level between 80 and 120 ng/ml. All the children were in the stage of complete remission of nephrotic syndrome at the time of the study. Apart from cyclosporine A patients enrolled into the study were treated with steroids at different doses; also they received antihistaminic drugs, proton pump inhibitors, and supplemented calcium, magnesium, and vitamin D. None of the patients enrolled into the study did not use ACE inhibitors at the time of the blood and urine samples collection.

All of the patients had cyclosporine C0 and C2 levels determined and they underwent biochemical tests as well to assess renal function (urea, creatinine, uric acid, and cystatin C concentrations) and tests of inflammation markers and biochemical parameters, which are typical disorders for nephrotic syndrome (serum protein concentration, lipid profile).

GFR from the serum creatinine concentration was calculated with the use of the Schwartz formula:

eGFR = *k*  × height [cm]/creatinine [mg/dl] (*k* = 0.55 for children aged 2–12 and girls up to the age of 18; *k* = 0.7 for boys aged 13–18).

GFR from the serum cystatin C concentration was calculated with the use of the Filler formula: eGFR = 1.962 × [1.123 × log(*l*/cys C)].

The concentration of cyclosporine A in the blood was determined using the ARCHITECT Cyclosporine test based on the immunochemical method with the use of microparticles and chemiluminescent marker (Chemiluminescent Microparticle Immunoassay (CMIA)). We collected a morning urine sample prior to the administration of CsA (NGAL 1) and another sample was gathered 2 to 4 hours after the CsA administration (NGAL 2) from all the patients to determine urine NGAL concentration. Urine samples collected for NGAL determination were frozen at −70 degrees Celsius. Enrollment of patients into the study and the collection of blood and urine samples lasted for six months.

After sample collection, the NGAL level was determined with the use of an immunoenzyme test by Bioporto Diagnostics, with strict adherence to the manufacturer's instructions. All samples were diluted to optimal density. The results of the immunoenzyme reaction were read with the use of a microplate spectrophotometer. The measurements were expressed as nanograms per millilitre. The limit of detection was 0.1 ng/ml.

The control group consisted of 23 children aged 4–17 (mean 10.1), including 14 boys and 9 girls, diagnosed in the Chorzow Centre for Paediatrics and Oncology due to monosymptomatic nocturnal enuresis. In the control group the first morning urine samples were obtained to measure NGAL and creatinine concentration and laboratory tests were performed to confirm a normal systemic metabolism.

The examination protocol was approved by the Bioethical Commission of the Medical University of Silesia, decision number 10/2009.

The statistical comparison of the study and control groups was preceded by the verification of continuous variable distributions with the normal distribution based on the Shapiro-Wilk test. For continuous variables with normal distribution, a parametric Student's *t*-test was used to analyse the differences between the groups.

For the variables not demonstrating normal distribution, a nonparametric Wilcoxon test was used.

To analyse the influence of the duration of cyclosporine A therapy on NGAL excretion and the influence of various factors on NGAL concentration changes and NGAL/creatinine ratio changes during CsA therapy, the *r*-Pearson correlation, Kendall Tau correlation, and linear robust regression models were used.

## 4. Results

The data presented in Table 2 demonstrates that the groups vary in median values of the analysed concentration of cystatin C and GFR calculated with Filler's formula. The study and control groups showed no differences in other features.

**Figure 1:**
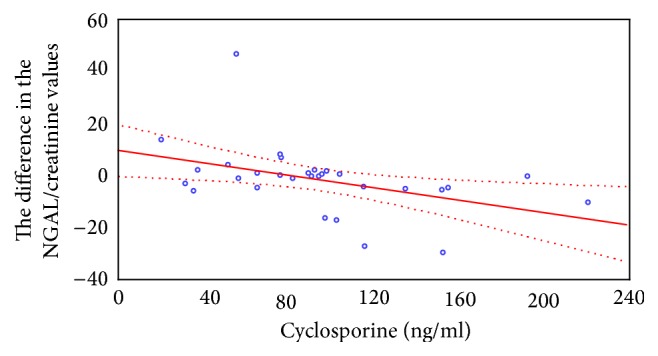
Correlation between the differences in NGAL/creatinine ratio in urine samples collected before and after CsA administration and C0 cyclosporine level.

A significant negative correlation was shown between the difference of the NGAL/creatinine ratio in urine samples collected before and after CsA administration and the C0 cyclosporine level. The higher the baseline concentration of the medicine, the lesser the influence of the next dose of the medicine on urinary NGAL excretion, expressed as a ratio of NGAL concentration to creatinine concentration. We found no statistically significant correlation between the C0 cyclosporine concentration and the difference of absolute urinary NGAL concentration expressed in ng/ml (Figure 1).

**Figure 2:**
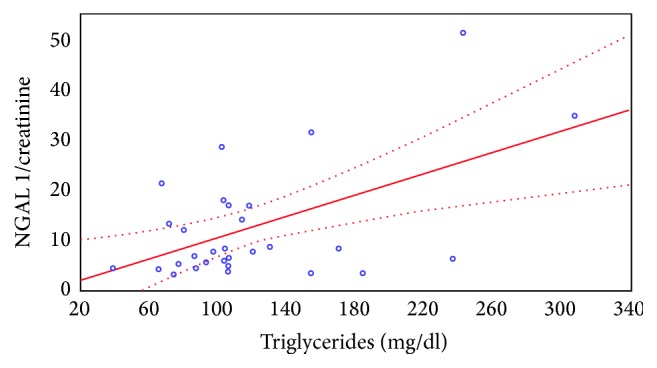
The correlation between the NGAL/creatinine ratio in the urine sample collected before CsA administration and the serum triglyceride level.

**Figure 3:**
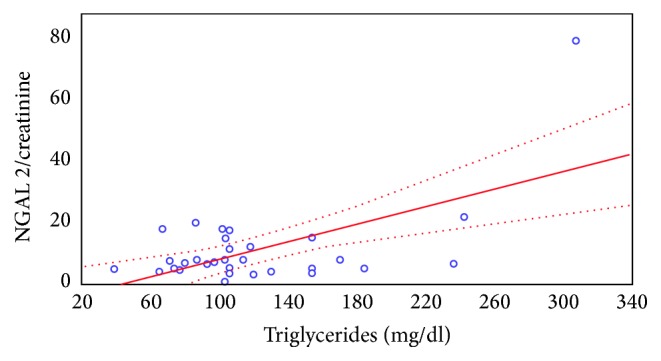
The correlation between the NGAL/creatinine ratio in the urine sample collected 2 hours after CsA administration and the serum triglyceride level.

Significant positive correlation was found between the NGAL/creatinine ratio in the urine samples and the serum concentration of triglyceride. The higher the serum triglyceride concentration, the higher the urinary NGAL excretion (Figures 2 and 3).

**Figure 4:**
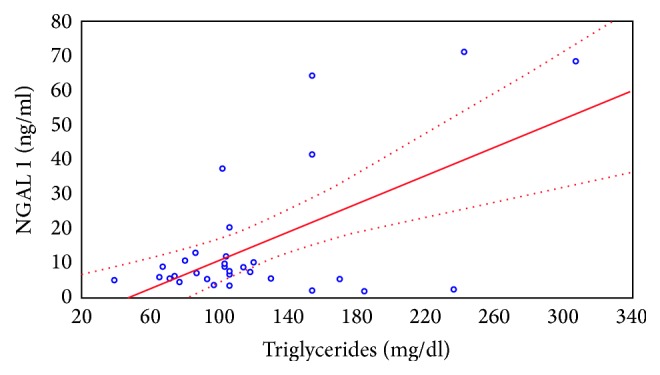
Relationship between NGAL concentration in the urine sample prior to CsA administration and serum triglyceride levels.

**Figure 5:**
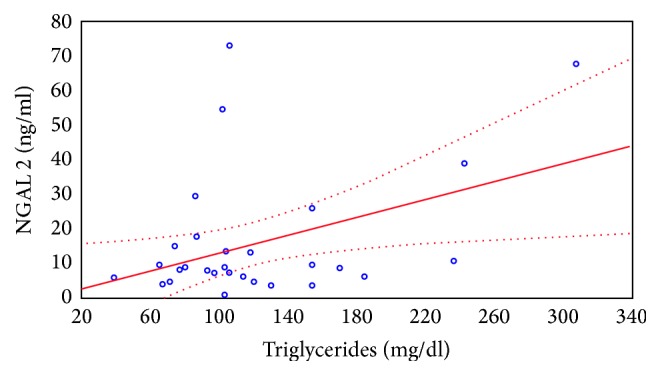
Relationship between NGAL concentration in the urine sample taken 2 hours after the CsA administration and serum triglyceride levels.

The similar dependence was observed between the absolute levels of NGAL excreted in urine expressed in ng/ml and the concentration of serum triglycerides. Relationship between NGAL concentration in the urine sample prior to and 2 hours after CsA administration and serum triglyceride levels is shown in Figures 4 and 5.

The statistical analyses performed using the regression robust model demonstrated an influence of the duration of cyclosporine A therapy on urinary NGAL excretion in the examined group.

**Figure 6:**
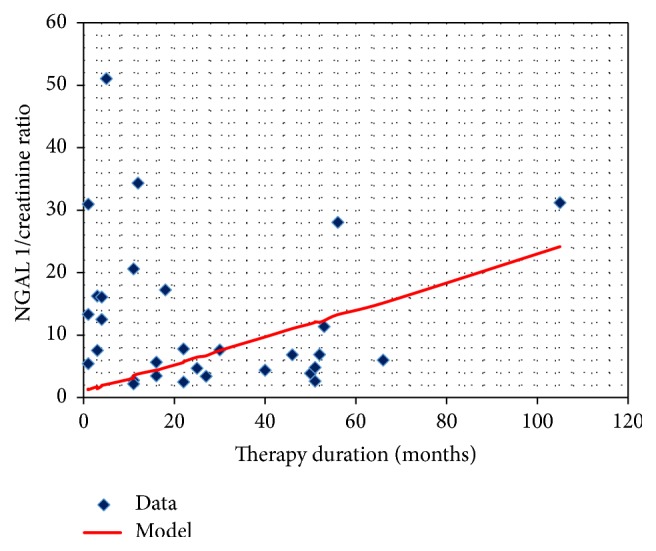
The NGAL/creatinine ratio in the urine sample collected before CsA administration and the duration of therapy.

The duration of treatment had a statistically significant influence on the NGAL to creatinine ratio in the urine sample collected before the administration of the drug. The concentration changes are presented in Figure 6.

**Figure 7:**
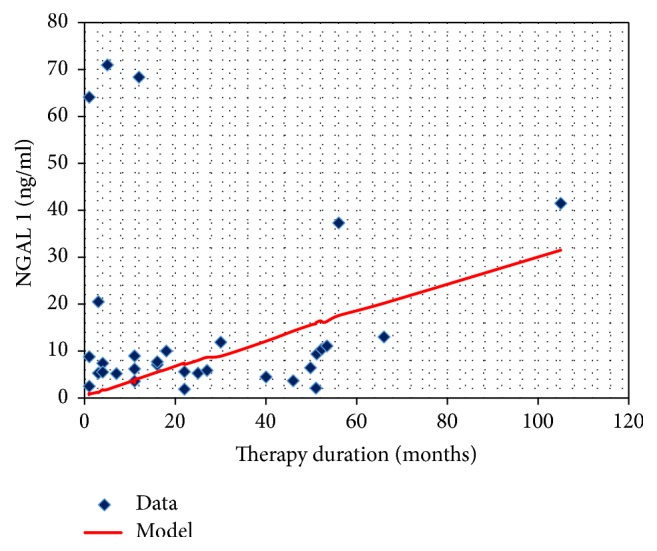
The NGAL concentration in the urine sample before CsA administration and the duration of therapy.

The similar dependence concerns NGAL urine excretion expressed in ng/ml (Figure 7).

**Figure 8:**
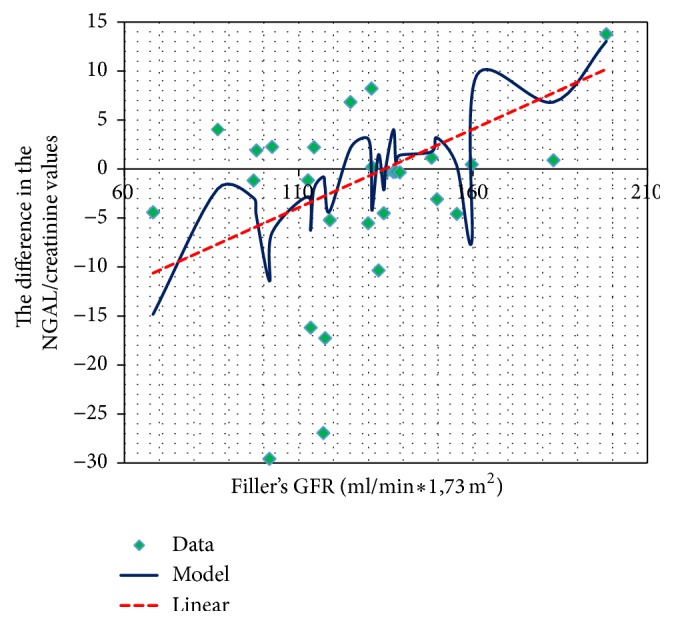
GFR and changes in the NGAL/creatinine concentrations before and after CsA administration.

The difference in the NGAL to creatinine ratio before and after the administration of CsA positively correlated with GFR calculated using the Filler formula (Figure 8).

Such a relationship with GFR calculated using Schwartz formula from the creatinine concentration was not observed.

**Figure 9:**
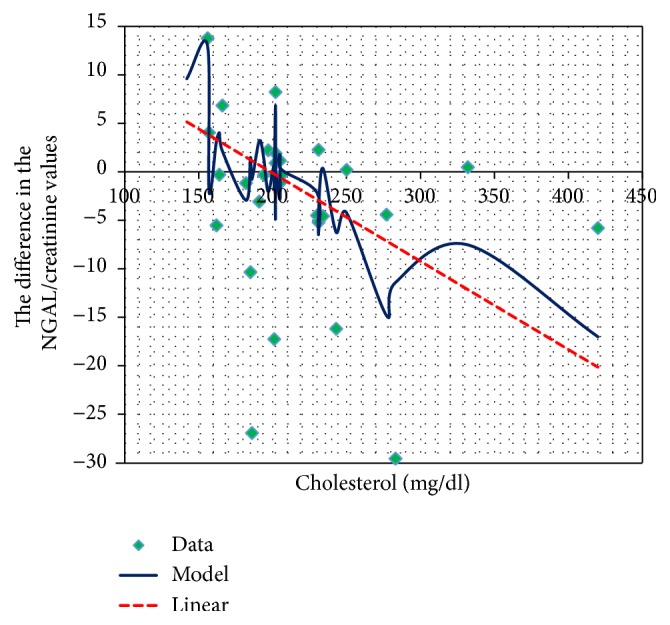
Total cholesterol and the changes in the NGAL/creatinine concentration before and after CsA use.

The difference in the NGAL to creatinine ratio before and after the CsA administration negatively correlated with total serum cholesterol concentration (Figure 9).

## 5. Discussion

In available literature there are only a few reports concerning the comportment of NGAL in children suffering from different types of nephrotic syndrome. It has been shown that in course of chronic glomerulonephritis plasma NGAL concentration and the urinary excretion of this protein are higher than in healthy people, but that does not apply to all types and periods of chronic glomerulonephritis [18–20]. Nishida et al. demonstrated that urine NGAL excretion is significantly increased during the period of nephrotic proteinuria and correlates with its severity and with reduction of estimated GFR calculated using the Schwartz method. The author points out that periods of remission of nephrotic syndrome are characterized by a low excretion of this biomarker, comparable to the control group [21].

Our research in children treated for various types of nephrotic syndrome demonstrated no differences in the mean concentration of NGAL in the urine samples compared to the control group. This may be due to the fact that all the children in the study group were in the remission period and in any case there was no significant proteinuria. This is essential for further consideration of cyclosporine's nephrotoxicity and the comportment of NGAL in children treated with this drug. It allows us to assume that NGAL in the urine is not a biomarker of only nephrotic syndrome. Cyclosporine A used in its treatment induces ischemia and the damage of the epithelial cells of renal tubules by the vasoconstriction of afferent arterioles. In experimental tests and the culture of human tubular cells in vitro it has been demonstrated that ischemic injury increases the NGAL mRNA [8, 22].

Because the nephrotoxic effects of many drugs, including CsA, are a huge clinical problem, attention was drawn to the potential usefulness of the NGAL urine concentration measurement, as an early biomarker of this complication [17, 23]. Sieber et al. demonstrated in the experimental study the increase of NGAL, KIM-1, clusterin, and TIMP-1 (tissue inhibitor of metalloproteinase) excretion in the detection of the nephrotoxic action of gentamicin. The authors presented a gentamicin dose dependent increase (60 versus 120 mg/kg) of urine NGAL and KIM-1 concentration before the onset of clinical signs of nephrotoxicity [24]. The increase of NGAL excretion was also demonstrated after intraperitoneal administration of cisplatin in mice.

To this date, there were very few reports in literature evaluating the usefulness of NGAL in the detection of nephrotoxicity of calcineurin inhibitors in patients after organ transplantation and those treated with other clinical indications. Wasilewska et al. conducted a survey concerning NGAL serum concentration and urinary NGAL excretion in children treated with cyclosporine A due to various types of nephrotic syndrome [17].

The study included 19 children with steroid-dependent nephrotic syndrome on the ground of MCD and FSGS. In the study group the serum concentrations of NGAL and NGAL excretion in the urine, expressed as a ratio of NGAL to creatinine in the urine sample, were measured four times. The first measurement was performed during exacerbation of nephrotic syndrome, prior to the initiation of CsA therapy. The subsequent measurements were performed in the 3rd, 6th, and 12th months of treatment.

The NGAL urine excretion differed between the study and the control group before the initiation of treatment. The difference increased up to 6 months of the therapy and after that the time differences in NGAL excretion were reduced, which may be connected with the reduction of cyclosporine dosage. In our study, we observed no differences in the urinary NGAL excretion between the study group and the control group.

The NGAL excretion in the urine in the study of Wasilewska et al. remained without any relation to the cyclosporine A blood concentration. This observation corresponds to our results.

In this study, the urinary NGAL excretion expressed as a ratio of NGAL to creatinine increased up to the 6th month of therapy and then decreased. In our observation, the urinary NGAL excretion converted per mg of creatinine increased in direct proportion to the duration of the cyclosporine treatment. The cyclosporine dependence, observed in a large number of patients, determines the elongation of CsA treatment. The treatment with this drug was initially predicted for a year or two. In many centers at the moment, patients are treated with cyclosporine even for several years. In this context increasing the concentrations of NGAL in urine during the treatment time observed in our study may be an important diagnostic clue indicating slow but steadily progressing renal tubular damage.

On one hand using CsA to treat nephrotic syndrome reduces lipid metabolism disorders, but on the other hand, it may itself cause hyperlipidemia. Cyclosporine as a lipophilic substance binds with lipoproteins in the plasma. Lipid disorders may therefore have an effect on drug metabolism and bioavailability. In examining the impact of various metabolic disorders in nephrotic syndrome on the urinary NGAL excretion in children treated with CsA, we found a strong, statistically significant positive correlation between NGAL and creatinine, calculated from both urine samples and the serum concentration of triglycerides. Moreno-Navarrete et al., investigating lipocalin 2 serum concentrations in patients with metabolic syndrome, insulin resistance, and excessive intake of fats, found a statistically significant positive correlation between changes in the concentration of triglycerides in the serum of subjects and changes in serum concentrations of NGAL [25]. Fassett et al. observed an effect of administration of atorvastatin on the serum concentration of lipocalin 2 [26]. There are no studies in available literature evaluating the effects of various lipid fractions concentration on the urine excretion of NGAL.

At the moment, lipocalin 2 aspires to the role of a “renal troponin.” Acute renal failure, as well as chronic kidney disease, is conditions that often coexist with the dysfunction of other organs and systems. A high proportion of patients diagnosed with renal diseases have serious lipid disorders. The influence of a concentration of serum triglycerides on the urinary NGAL excretion found in our study certainly requires further research and maybe it will be included in the interpretation of tests in the future.

In our study we found a negative correlation between the total cholesterol level and the difference in the NGAL urine concentration in the samples collected before and 2 hours after the administration of CsA. The theoretically high concentration of CsA two hours after the administration of the drug should stimulate ischemic nephron injury, which would enhance lipocalin excretion by the proximal tubule and collecting duct cells. However, our study did not reveal such a correlation, which seems to be connected with very high cholesterol levels in children with nephrotic syndrome. Cyclosporine as an active lipophilic substance binds with lipoproteins present in excess in the serum of sick children. Perhaps for this reason, its vasoconstrictive reaction in these children is not as rapid as expected.

The difference in urinary NGAL excretion before the administration of CsA and at the peak of its serum concentration in the examined children demonstrated a positive correlation with the GFR calculated using the Filler method (but not the Schwartz method).

The results of our study demonstrate that the concentration of NGAL in urine (expressed as a NGAL to creatinine ratio in the urine sample) cannot be used to detect early stages of CsA nephrotoxicity in children with nephrotic syndrome that is treated with this drug. This observation is consistent with evidence obtained from the only available study in literature evaluating NGAL as a potential marker of CsA nephrotoxicity in children with nephrotic syndrome. The complex correlation between the urine lipocalin excretion and characteristic for the nephrotic syndrome metabolic disorders complicates the interpretation of the results. The positive correlation between the urine NGAL concentration over the course of the CsA treatment seems to have a big practical meaning in the context of prolonged CsA treatment observed in recent years.

The examination of the urinary NGAL concentration is a simple and noninvasive test delivering certain information concerning the condition of renal parenchyma. Its periodic measurement and interpretation together with other available parameters of nephrotoxicity may be beneficial in the individualization and optimization of CsA therapy.

## 6. Conclusions

1. The concentration of NGAL in urine cannot act as an early detector of CsA nephrotoxicity in children with nephrotic syndrome treated with this drug.
2. The concentration of NGAL excreted in the urine demonstrates a statistically significant correlation with serum triglycerides concentration.
3. A statistically significant correlation between the urinary NGAL concentration and the duration of the CsA treatment indicates the potential usefulness of this marker in the monitoring of CsA nephrotoxicity, but only in relation to the other available indicators of kidney function.

## Figures and Tables

**Table 1:** Characteristics of the examined group.

Number	Age	Sex	Duration of disease (years)	Total CsA therapy duration (months)	Indications for CsA introduction	Histopathological findings
1	17	F	5	3	Steroid-resistant NS	Mesangial proliferative GN (glomerulonephritis)
2	17	M	2	3	Steroid-resistant NS	Generalized and segmental fibrosis
3	17	M	14	51	Alport syndrome	Alport syndrome (fibrosis, foam cells)
4	16	M	14	30	Steroid-dependent NS	Mesangial proliferative GN
5	15	M	13	16	Alport syndrome	Alport syndrome (foam cells)
6	14	F	11	66	Steroid-dependent NS	Minimal changes
7	13	M	10	50	Steroid-dependent NS	Minimal changes with tendency to FSGS (focal segmental glomerulosclerosis)
8	13	M	11	22	Steroid-resistant NS	IgA nephropathy
9	13	F	2	12	Steroid-dependent NS	Minimal changes with tendency to FSGS
10	12	M	10	105	Steroid-dependent NS	None
11	12	M	10	51	Steroid-dependent NS	Minimal changes
12	11	F	6/12	2	Steroid-resistant NS	Membranoproliferative GN
13	11	F	3	6	Steroid-dependent NS	None
14	10	M	6	52	Steroid-resistant NS	Minimal changes
15	9	M	7	56	Steroid-resistant NS	Minimal changes
16	9	M	5	53	Steroid-resistant NS	FSGS
17	7	F	4	40	Steroid-dependent NS	None
18	7	F	5	11	Steroid-dependent NS	None
19	7	M	1	7	Steroid-resistant NS	Minimal changes
20	6	M	4	25	Steroid-dependent NS	Minimal changes/mesangial proliferative GN
21	6	M	5	46	Steroid-resistant NS	Minimal changes
22	5.5	M	3	11	Steroid-dependent NS	None
23	5	M	3	27	Steroid-dependent NS	None
24	4.5	F	3	22	Steroid-dependent NS	None
25	4.5	F	2	3	Steroid-resistant NS	Membranoproliferative GN
26	4.5	M	3	16	Steroid-dependent NS	None
27	4	M	2	18	Steroid-dependent NS	None
28	4	M	3	4	Steroid-dependent NS	None
29	4	F	1	11	Steroid-resistant NS	None
30	3	F	5/12	3	Steroid-resistant NS	None
31	3	F	1	4	Steroid-dependent NS	None

**Table 2:** Comparison of the study and control groups in terms of parameters not demonstrating normal distribution (Wilcoxon test).

Feature	Examined group	Control group	W statistics	*p* value
NGAL 1/creatinine [ng/mg]	7.22 (4.45; 16.23)	6.48 (3.25; 14.01)	282	0.2645
Cystatin C [mg/l]	0.73 (0.69; 0.83)	0.58 (0.44; 0.65)	77	<0.0001
GFR according to Schwartz [ml/min/1.73 m^2^]	125.3 (113.95; 139)	124.1 (115.5; 135.6)	344.5	0.7301
GFR according to Filler [ml/min/1.73 m^2^]	130.87 (113.41; 138.99)	169.24 (149.8; 230.25)	590	<0.0001
